# Efficacy and safety of neoadjuvant immunotherapy in resectable esophageal or gastroesophageal junction carcinoma: A pooled analysis of prospective clinical trials

**DOI:** 10.3389/fimmu.2022.1041233

**Published:** 2022-12-16

**Authors:** Jie Zhu, Xuefeng Leng, Binyang Gao, Bo Wang, Hanlin Zhang, Lei Wu, Jiabao Ma, Yan Tan, Lin Peng, Yongtao Han, Qifeng Wang

**Affiliations:** ^1^ Radiation Oncology Key Laboratory of Sichuan Province, Department of Radiation Oncology, Sichuan Cancer Hospital & Institute, Sichuan Cancer Center, School of Medicine, University of Electronic Science and Technology of China, Chengdu, China; ^2^ Department of Thoracic Surgery, Sichuan Cancer Hospital & Institute, Sichuan Cancer Center, School of Medicine, University of Electronic Science and Technology of China, Chengdu, China; ^3^ Department of Ultrasound, West China Hospital of Sichuan University, Chengdu, China; ^4^ Kidney Research Institute, Department of Nephrology, West China Hospital of Sichuan University, Chengdu, China; ^5^ Department of Dermatology, Peking Union Medical College Hospital, Chinese Academy of Medical Sciences and Peking Union Medical College, Beijing, China

**Keywords:** neoadjuvant, immunotherapy, immunochemoradiotherapy, immune checkpoint inhibitor, resectable esophageal carcinoma

## Abstract

Neoadjuvant chemoradiotherapy (NCRT) plus radical esophagectomy is currently the standard treatment for resectable esophageal or gastroesophageal junction (GEJ) carcinoma. The aim of this study is to evaluate the efficacy and safety of neoadjuvant immunotherapy in resectable esophageal or GEJ carcinoma. Prospective clinical trials investigating efficacy and/or safety of neoadjuvant immunotherapy with immune checkpoint inhibitors (ICIs) followed by radical esophagectomy in patients with newly diagnosed resectable esophageal or GEJ carcinoma were identified through literature search. Quality assessment was performed by using the Newcastle–Ottawa scale. Preliminary treatment outcomes of pathologically complete response (pCR, ypT0N0) and grade 3-4 adverse effects (AEs) were pooled together and then compared with standard NCRT of the historical control CROSS study by Chi-square (χ^2^) test. A two-sided *P* value < 0.05 was considered statistically significant. A total of 17 eligible non-randomized trials with 455 participants were included into analysis. The most common primary endpoint was pCR (n = 7, 41%), and the median sample size and follow-up period was 23 patients and 7.9 months, respectively. For patients receiving neoadjuvant immunotherapy, the overall pCR, R0 resection, and grade 3-4 AE rates were 33.2%, 95.5%, and 35.1%, respectively. For esophageal squamous cell carcinoma (ESCC) and adenocarcinoma (EAC), neoadjuvant immunochemoradiotherapy showed no significant improvement in pCR rate than NCRT (ESCC, 50% vs 48.7%, *P* = 0.9; EAC, 32.6% vs 23.1%, *P* = 0.22). Grade 3-4 AEs were the most common in patients with neoadjuvant immunochemoradiotherapy, significantly higher than immunochemotherapy (46.7% vs 32.8%, *P* = 0.04) and NCRT (46.7% vs 18.1%, *P* < 0.0001). In conclusion, for patients with resectable esophageal or GEJ carcinoma, the addition of ICIs to standard NCRT could not improve pCR rate in both ESCC and EAC, but significantly increased the risk of severe AEs. Large-scale phase 3 randomized trials were urgently needed to further confirm the survival benefit and safety profile of neoadjuvant immunotherapy.

## Introduction

Esophageal or gastroesophageal junction (GEJ) carcinoma is the sixth most common cancer-related cause of death worldwide, with an estimated 500,000 deaths annually (1). Currently, multimodal treatment of neoadjuvant chemoradiotherapy (NCRT) plus radical esophagectomy has been the standard treatment for resectable esophageal or GEJ carcinoma (2). However, due to a high risk of disease progression and distant metastasis, the long-term survival remains dismal for regionally advanced stage, with a 5-year overall survival (OS) of 25% (3, 4). It is urgent to develop more effective treatment strategies to prolong survival of patients with resectable esophageal or GEJ carcinoma.

Immune checkpoint inhibitors (ICIs), as a monotherapy or in various combinations, have revolutionized anti-cancer therapy in many solid tumors. For advanced/metastatic esophageal or GEJ carcinoma, programmed cell death receptor 1 (PD-1) inhibitors pembrolizumab and nivolumab have significantly prolonged survival than conventional chemotherapy regimens (5–7). In this direction, a wide range of ICIs is currently under investigation in locally advanced esophageal or GEJ carcinoma. The large-scale phase 3 randomized controlled trial (RCT) CheckMate 577 showed that postoperative nivolumab maintenance in resected (R0) stage II or III esophageal or GEJ carcinoma after standard NCRT significantly reduced the risk of recurrence or death by 31% (8). In the setting of neoadjuvant therapy with ICIs, no results from phase 3 RCT have been formally reported so far. Prospective treatment outcomes of neoadjuvant immunotherapy in resectable esophageal or GEJ carcinoma were only investigated in small-scale phase 1 or 2 non-randomized clinical trials (9–11), with ambiguous and inaccurate efficacy outcomes and safety profiles due to limited sample size. A recently published real-world retrospective study mainly investigating neoadjuvant immunochemotherapy in resectable esophageal or GEJ carcinoma showed a pathologically complete response (pCR) rate of 25.8% (ypT0N0) and acceptable safety profile (12). It is worth noting that real-world outcomes, especially safety profiles, may be potentially confounded by selection biases and incomplete medical records. Generally, clinical trials have more comprehensive quality control and are often regarded as high-level evidence. Therefore, it is necessary to further assess the efficacy and safety of neoadjuvant immunotherapy through pooling prospective trials together.

In this study, we performed a pooled analysis of prospective clinical trials of resectable esophageal or GEJ carcinoma treated with neoadjuvant immunotherapy. The primary purpose of this study is to compare the efficacy and safety of neoadjuvant immunotherapy with standard NCRT.

## Materials and methods

### Inclusion and exclusion criteria

This study was exempted from review by the institutional review board because it only collected published data, and no human subjects were newly enrolled. This study included prospective clinical trials investigating efficacy and/or safety of neoadjuvant immunotherapy with ICIs followed by radical esophagectomy in patients with newly diagnosed resectable esophageal or GEJ carcinoma. Studies were excluded if they met any of the followings: retrospective cohort study, inoperable patients, previously treated patients, conventional neoadjuvant chemotherapy/radiotherapy, inadequate report on efficacy and safety, non-epithelial histology (e.g., lymphomas or sarcomas), or non-English publication.

### Literature search

Clinical trials published before June 2022 were identified through a systematic literature search of Embase, PubMed, Web of Science, and the Cochrane Library by using the following search terms: (“esophageal” or “esophagus” or “esophagogastric” or “gastroesophageal”) AND (“neoadjuvant” or “preoperative”) AND (“immunotherapy” or “immunochemotherapy” or “immunochemoradiotherapy” or “PD-1” or “PD-L1” or “pembrolizumab” or “nivolumab” or “atezolizumab” or “camrelizumab” or “sintilimab” or “toripalimab”), with article type restricted to prospective study. A manual search on article references and abstracts of American Society of Clinical Oncology (ASCO) and European Society for Medical Oncology (ESMO) annual meetings was also performed to include potentially eligible studies. The literature search and study inclusion were independently conducted by JZ and BG, and disagreements were further resolved by the consensus of JZ, BG, and QW.

### Quality assessment

For non-randomized trials or cohort studies, quality was assessed with a maximum 9-star score, by using the Newcastle–Ottawa scale (NOS) in terms of selection, comparability, and outcome (13). Trials with low to moderate risk of bias (≥ 6 stars) were included in the statistical analysis, and those with high risk of bias (< 6 stars) were excluded. All information available in the assessment was acquired from formal publications, meeting abstracts, and trial registry information on ClinicalTrials.gov (https://www.clinicaltrials.gov).

### Endpoint definition

The primary endpoint pCR was defined as no residual cancer cells in both tumor and lymph node (ypT0N0). R0 resection was defined as no cancer cells at resection margins microscopically. Adverse effects (AEs) were graded according to the criteria of original studies, mostly the National Cancer Institute Common Terminology Criteria for Adverse Events (CTCAE).

### Data extraction

Data including trial phase, pathological type, primary endpoint, median follow-up time, sample size, neoadjuvant treatment regimen, pCR rate, R0 resection rate, grade 3-4 AE rate, and CTCAE version were extracted.

### Statistical analysis

The CROSS study was taken as the historical control, because the chemoradiotherapy schedule of 4 trials investigating neoadjuvant immunochemoradiotherapy was the same as the CROSS study, which included carboplatin (area under the curve of 2 mg per milliliter per minute, once a week for 5 weeks), paclitaxel (50 mg/m^2^, once a week for 5 weeks), and radiotherapy (23 fractions of 1.8 Gy, 5 fraction a week) (9–11, 14).

Overall efficacy and safety were calculated after pooling results of all trials. Chi-square (χ^2^) test was used to compare treatment outcomes between different neoadjuvant treatment regimens. Subgroup analysis was conducted to investigate the efficacy and safety of different chemotherapy schemes. To assess the consistency and robustness of the overall efficacy and safety across different settings, sensitivity analysis was performed by leaving each trial out at a time. Statistical analysis was performed by SPSS statistical software (version 21.0, Armonk, NY: IBM Corp.). A two-sided *P* value < 0.05 was considered statistically significant.

## Results

**Figure 1:**
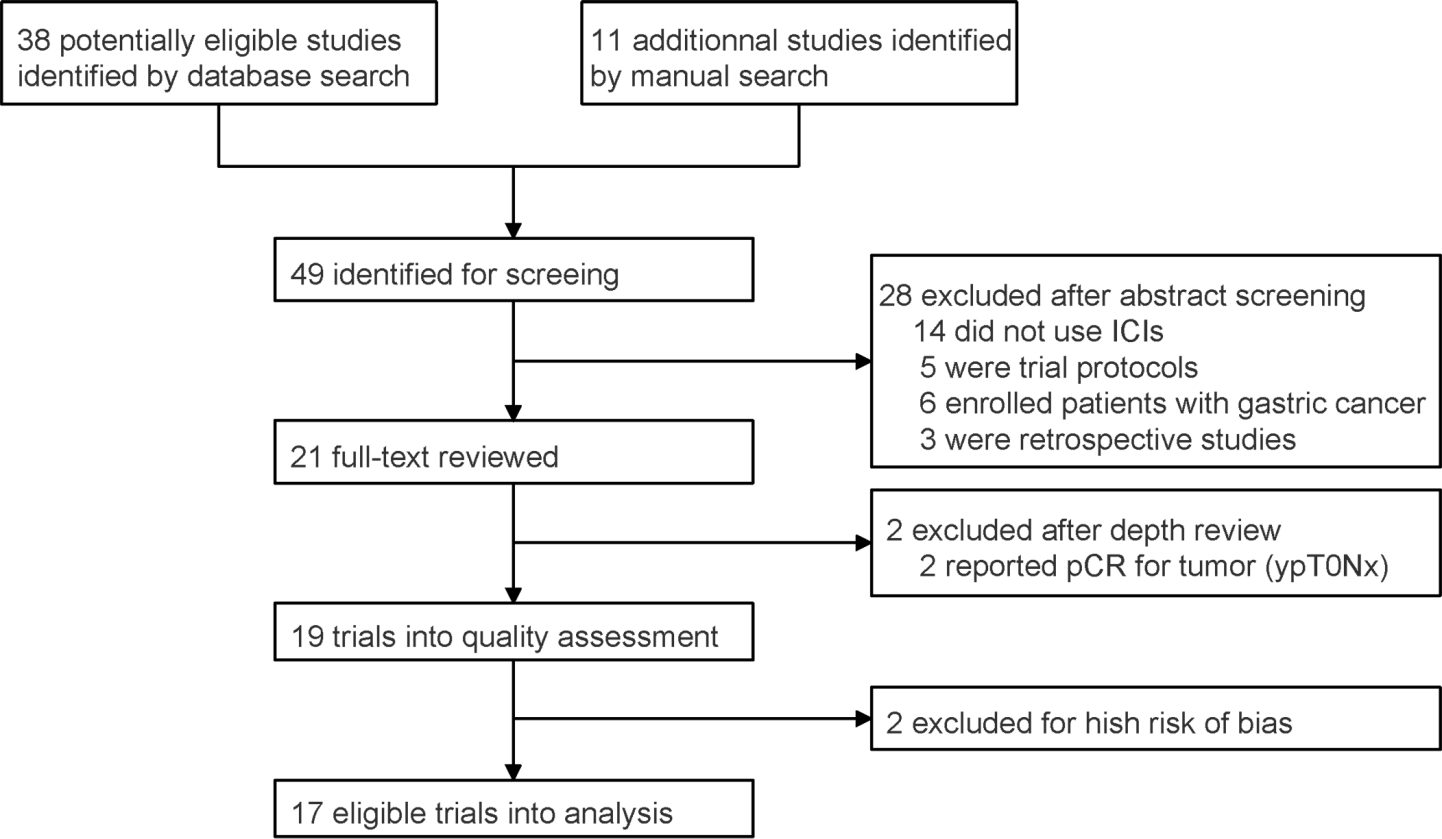
PRISMA flow chart for prospective trial inclusion. ICI, immune checkpoint inhibitor; pCR, pathologically complete response.

A total of 49 studies were identified after database and manual searches. Twenty-eight studies were excluded after abstract screening. ESONICT-2 (15) and NIC-ESCC2019 (16) trials were not included into efficacy analysis after depth review, because only pCR for primary tumor (ypT0Nx) was reported in these 2 studies and the regression of lymph nodes was unclear. After quality assessment, trials NCT03917966 (17) and NICE (18) were further excluded for high risk of bias, because they did not finish recruitment or follow-up (**Supplemental Table 1**). Eventually, 17 eligible trials with 455 participants were enrolled into analysis (**Figure 1**; **Table 1**) (9–11, 14, 19–32).

**Table 1:** Summary of the trials of neoadjuvant immunotherapy for resectable esophageal or gastroesophageal junction carcinoma.

Trial	Phase	Pathology	Primaryendpoint	MedianFU	No.	Neoadjuvant treatment	pCR	R0 resection	AEs	
									Grade 3-4 (%)	CTCAE version
Neoadjuvant immunochemoradiotherapy (n = 4)										
PALACE-1 (Li, 2021)^9^	1	SCC	Safety	NA	20	Pembrolizumab + CROSS schedule	10/18	NA	12/20	4.03
PERFECT (van den Ende, 2021)^10^	2	AC	Feasibility	NA	40	Atezolizumab + CROSS schedule	10/33	33/33	16/40	4.03
NCT03044613 (Kelly, 2019)^11^	1	AC	Safety, feasibility	NA	16	Nivolumab + CROSS schedule	4/10	NA	NA	4.03
NCT02844075 (Lee, 2019)^14^	2	SCC	pCR	12.4m	28	Pembrolizumab maintenance + CROSS schedule	12/26	NA	NA	4.03
Neoadjuvant immunochemotherapy (n = 13)										
ChiCTR2000028900 (Yang, 2022)^19^	1	SCC	Safety, feasibility	13.8m	23	Camrelizumab + TC*	5/20	20/20	11/23	5.0
TD-NICE (Yan, 2022)^20^	2	SCC	MPR	NA	45	Tislelizumab + TC*	18/36	29/36	19/45	NA
NCT04177797 (He, 2022)^21^	2	SCC	Safety, feasibility, and MPR	NA	20	Toripalimab + TC	3/16	14/16	4/20	4.03
ChiCTR1900026240 (Liu, 2022)^22^	2	SCC	pCR	NA	60	Camrelizumab + TC*	20/51	50/51	34/60	5.0
NCT03985670 (Xing, 2021)^23^	2	SCC	pCR	NA	15	Toripalimab (day 3) + TP (day 1)	4/11	11/11	3/15	5.0
					15	Toripalimab (day 1) + TP (day 1)	1/13	13/13	7/15	
ESONICT-1 (Zhang, 2021)^24^	2	SCC	pCR, AEs	6m	30	Sintilimab + nab-paclitaxel + cisplatin	4/23	23/23	1/30	5.0
Shen, 2021^25^	NA	SCC	Safety, feasibility	6m	28	PD-1 inhibitor + TC*	9/27	26/27	2/28	5.0
Yang, 2021^26^	NA	SCC	pCR	NA	16	Camrelizumab + TC	5/16	15/16	NA	5.0
SIN-ICE (Duan, 2021)^27^	NA	SCC	pCR	NA	23	Sintilimab + platinum-based chemotherapy	6/17	16/17	7/23	4.03
KEEP-G 03 (Gu, 2020)^28^	1/2	SCC	Safety, feasibility	NA	17	Sintilimab + lipo-paclitaxel + cisplatin + S-1	4/15	15/15	6/17	5.0
Zhang, 2020^29^	2	SCC	MPR	7.9m	24	Toripalimab + nab-paclitaxel + S-1	3/18	NA	NA	NA
Li, 2020^30^	2	SCC	pCR, MPR	4.5m	17	Toripalimab + TC*	2/12	12/12	2/17	NA
FRONTiER (Yamamoto, 2021; Matsuda, 2022)^31,32^	1	SCC	Toxicities	NA	6	Nivolumab + CF (cohort A)	2/6	6/6	NA	4.03
					12	Nivolumab + DCF (cohort C and D)	4/12	11/12	NA	
Historical control NCRT (n = 1)										
CROSS (Shapiro, 2015)^2^	3	SCC	OS	84m	41	TC + RT	18/37	146/161	31/171	3.0
		AC			134		28/121			

AC, adenocarcinoma; AE, adverse effect; CF, cisplatin plus fluorouracil; DCF, docetaxel, cisplatin plus fluorouracil; FU, follow-up; MPR, major pathological response; NA, not available; NCI-CTCAE, National Cancer Institute-Common Toxicity Criteria for Adverse Events; OS, overall survival; pCR, pathologically complete response; RT, radiotherapy; SCC, squamous cell carcinoma; TC, paclitaxel plus carboplatin; TC*, nab-paclitaxel plus carboplatin.

A majority of studies were non-randomized phase 2 clinical trials (n = 10, 59%), with no RCTs included. The most common primary endpoint was pCR (n = 7, 41%), followed by safety (n = 6, 35%), feasibility (n = 6, 35%), and MPR (n = 4, 24%). The median sample size and follow-up period was 23 patients and 7.9 months, respectively. There were 104 (21%) and 351 (77%) participants receiving neoadjuvant immunochemoradiotherapy and immunochemotherapy, respectively. There were 399 (88%) and 56 (12%) patients with the pathological type of esophageal squamous cell carcinoma (ESCC) and esophageal adenocarcinoma (EAC), respectively. For all patients receiving neoadjuvant immunotherapy, the overall pCR, R0 resection, and grade 3-4 AE rates were 33.2%, 95.5%, and 35.1%, respectively.

**Figure 2:**
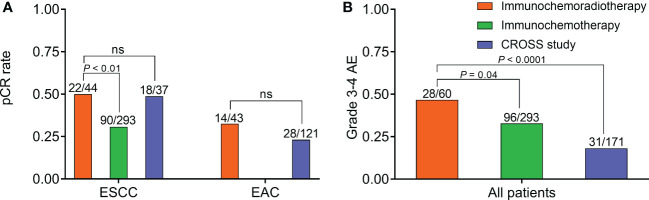
Efficacy and safety of neoadjuvant immunochemoradiotherapy, neoadjuvant immunochemotherapy, and CROSS study. **(A)** Comparison of pCR rate in ESCC and EAC, respectively. **(B)** Comparison of grade 3-4 AE rate in all patients. AE, adverse effect; EAC, adenocarcinoma; ESCC, esophageal squamous cell carcinoma; NS, not significant; pCR, pathologically complete response.

For ESCC, neoadjuvant immunochemoradiotherapy showed no significant improvement in pCR rate than NCRT of the CROSS study (50% vs 48.7%, *P* = 0.9), but demonstrated a significantly higher pCR rate than immunochemotherapy (50% vs 30.7%, *P* < 0.01). A significantly lower pCR rate in ESCC was observed after neoadjuvant immunochemotherapy than NCRT (30.7% vs 48.7%, *P* = 0.04) (**Figure 2**). For EAC, there were no significant difference in pCR rate between neoadjuvant immunochemoradiotherapy and NCRT (32.6% vs 23.1%, *P* = 0.22) (**Figure 2**). For patients with neoadjuvant immunochemoradiotherapy, there was a tendency of improved pCR rate in pathological type ESCC than EAC, but without statistical significance (50% vs 32.6%, *P* = 0.1). Sensitivity analysis of 13 trials receiving neoadjuvant immunochemotherapy demonstrated acceptable consistency with no significant differences in overall pCR and R0 resection rate (*P* > 0.05) (**Supplemental Figure 1**).

In safety assessment, 7 (41%), 7 (41%), and 3 (18%) trials applied CTCAE v4.03, CTCAE v5.0, and unknown criteria, respectively. The historical control CROSS study adopted CTCAE v3.0. Grade 3-4 AEs were the most common in patients with neoadjuvant immunochemoradiotherapy, significantly higher than immunochemotherapy (46.7% vs 32.8%, *P* = 0.04) and NCRT (46.7% vs 18.1%, *P* < 0.0001) (**Figure 2**). Besides, one patient died from esophageal hemorrhage after receiving neoadjuvant immunochemoradiotherapy in PALACE-1 trial (9). Sensitivity analysis of neoadjuvant immunochemotherapy showed acceptable consistency in grade 3-4 AEs (*P* > 0.05) (**Supplemental Figure 1**).

In subgroup analysis of neoadjuvant immunochemotherapy in ESCC, trials with chemotherapy regimen paclitaxel or nab-paclitaxel plus carboplatin, every 3 weeks (TC scheme) were included. Overall pCR, R0 resection, and grade 3-4 AE rates were 34.8%, 93.2%, and 37.3%, respectively. Neoadjuvant immunochemoradiotherapy demonstrated no significant improvement in pCR rate (50% vs 34.8%, *P* = 0.06) and grade 3-4 AEs (46.7% vs 37.8%, *P* = 0.20) than neoadjuvant immunochemotherapy with TC scheme. With the cost of significantly increased grade 3-4 AEs (37.3% vs 18.1%, *P* < 0.0001), neoadjuvant immunochemotherapy with TC scheme failed to show a superior pCR outcome than CROSS trial (34.8% vs 48.7%, *P* = 0.14). For trials with neoadjuvant immunochemotherapy of cisplatin-based regimens in ESCC, overall pCR, R0 resection, and grade 3-4 AE rates were 25.8%, 97.9%, and 24%, respectively. The cisplatin-based regimens exhibited superior safety than TC regimen in neoadjuvant immunochemotherapy (grade 3-4 AE rate: 24% vs 37.3%, *P* = 0.02).

## Discussion

This pooled analysis enrolling qualified prospective trials of resectable esophageal or GEJ carcinoma treated with neoadjuvant immunotherapy indicated that preoperative addition of ICIs to standard NCRT could increase the risk of severe AEs, however, without significant improvement in pCR. These findings provided more objective and comprehensive efficacy and safety evaluation of neoadjuvant immunotherapy than single small-scale trial, which could help with precise sample size calculation in future trials and accelerate the process of phase 3 RCTs.

A recently published real-world multicenter retrospective study in China which mainly included patients with locally advanced ESCC reported a pCR rate of 42.3% and 25.5% after neoadjuvant immunochemoradiotherapy and immunochemotherapy, respectively, which was approximative to the prospective outcomes in this study (12). In standard NCRT, the addition of radiotherapy to chemotherapy could significantly promote tumor shrinkage (33). The application of concurrent radiotherapy to immunochemotherapy could further facilitate tumor antigens exposure and enhance diverse immune response (34), therefore, compared with immunochemotherapy, improved pCR resection rate was expected in ESCC patients with neoadjuvant immunochemoradiotherapy. Although the addition of ICIs to standard NCRT did not improve pCR rate in this study, it was likely that patients once achieving pCR after immunotherapy had the potential to acquire a prolonged disease-free survival, because the duration of response was substantially longer in immunotherapy compared with conventional chemotherapy (35). The median time to response was 4.1 months for patients with advanced or metastatic esophageal cancer receiving pembrolizumab monotherapy (35), and the total duration from the start of standard NCRT with CROSS schedule to radical esophagectomy was 2 to 2.5 months (2), which may be not long enough for ICIs to evoke effective immune response in current trial settings. Considering the characteristics of delayed immune activation and prolonged duration of response in immunotherapy, explorations on extended use of ICIs before and/or after chemoradiotherapy and delayed surgery were highly encouraged in phase 3 RCTs.

For safety, it should be cautious that the addition of ICIs to NCRT could increase the risk of severe even lethal complications. Compared with this pooled analysis of trials, grade 3-4 AEs were less frequently reported in the real-world practice, with 23.3% and 12.7% in neoadjuvant immunochemoradiotherapy and immunochemotherapy, respectively (12). The disagreements on safety evaluation may resulted from the different nature of two studies, and we tended to hold the opinion that severe AEs were underestimated in real-world data possibly due to non-standardized medical records and potential selective preference to patients with good performance or few comorbidities. It was well recognized that the long-term survival was determined by both treatment effect and toxicity. For patients with resectable esophageal or GEJ carcinoma treated with standard NCRT, the overall toxicity was acceptable, and the achievement on tumor response was successfully converted to survival prolongation (36). However, for patients treated with neoadjuvant immunotherapy, frequently occurred severe complications might partly diminish the potential survival benefit from immunotherapy, which needed to be confirmed by large-scale RCTs with long follow-up. Particularly, immune-related death occurred in patients with neoadjuvant immunochemoradiotherapy mainly due to esophageal bleeding or pneumonitis (12). To decrease the risk of lethal complications, radiation oncologists could consider the feasibility of reducing radiation dose, shrinking target area, and involving field irradiation when radiotherapy and ICIs were combined.

There were some limitations in this study. First, this is a literature-based pooled analysis with individual patient data unavailable, thus patient-level safety profile was absent. The report on AE in this study was mainly about incidence, not comprehensive safety profile. Patient-level safety profile should be comprehensively evaluated in further research by using individual patient data. Second, the follow-up time of enrolled trials was not long enough for adequate report on long-term survival, and the survival benefit was unable to evaluate in this study. Third, from 2006 to 2017, the CTCAE criteria were evolved from v3.0 to v5.0. CTCAE v3.0 mainly assessed objective tissue damage and clinical examinations. CTCAE v4.03 further evaluated symptoms and functional abnormalities additionally, and showed advantages over v3.0 in delegating quality of life. Compared with CTCAE v4.03, v5.0 mainly updated 54 algorithms in 19 lab parameters, with few changes in grade criteria. The heterogeneity in AE assessment existed and could not be removed in this study.

In conclusion, for patients with resectable esophageal or GEJ carcinoma, the addition of ICIs to standard NCRT could not improve pCR rate in both pathological types ESCC and EAC, but significantly increased the risk of severe AEs. Large-scale phase 3 randomized trials were urgently needed to further confirm the long-term survival benefit of neoadjuvant immunotherapy.

## Data availability statement

The original contributions presented in the study are included in the article/**Supplementary Material**. Further inquiries can be directed to the corresponding authors.

## Ethics statement

Ethical review and approval was not required for the study on human participants in accordance with the local legislation and institutional requirements. Written informed consent for participation was not required for this study in accordance with the national legislation and the institutional requirements.

## Author contributions

QW, YH, and LP designed the study and revised the manuscript. JZ collected raw data, performed statistical analysis, and drafted the manuscript. JZ and BG performed literature search. XL, BW, LW, HZ, JM, and YT collected raw data. All authors contributed to the article and approved the submitted version.
