# Supplementary material for: Efficacy and safety of neoadjuvant immunotherapy in resectable esophageal or gastroesophageal junction carcinoma: A pooled analysis of prospective clinical trials
Source: Front Immunol. 2022 Dec 16;13:1041233. doi: 10.3389/fimmu.2022.1041233 (PMC9800859; doi:10.3389/fimmu.2022.1041233)
Supplement: Supplementary file 1 [file DataSheet_1.docx]

| **Supplemental Table 1.** Newcastle–Ottawa quality assessment scale for non-randomized controlled trials | | | | | | | | | |
| --- | --- | --- | --- | --- | --- | --- | --- | --- | --- |
| Trial | Selection | | | | Comparability | Outcome | | | Total score |
|  | Item 1 | Item 2 | Item 3 | Item 4 | Item 1 | Item 1 | Item 2 | Item 3 |  |
| PALACE-1 (Li, 2021) | 1 | 0 | 1 | 1 | 0 | 1 | 1 | 1 | 6 |
| PERFECT (van den Ende, 2021) | 1 | 1 | 1 | 1 | 2 | 1 | 1 | 1 | 9 |
| NCT03044613 (Kelly, 2019) | 1 | 0 | 1 | 1 | 0 | 1 | 1 | 1 | 6 |
| NCT02844075 (Lee, 2019) | 1 | 0 | 1 | 1 | 0 | 1 | 1 | 1 | 6 |
| ChiCTR2000028900 (Yang, 2022) | 1 | 0 | 1 | 1 | 0 | 1 | 1 | 1 | 6 |
| TD-NICE (Yan, 2022) | 1 | 0 | 1 | 1 | 0 | 1 | 1 | 1 | 6 |
| NCT04177797 (He, 2022) | 1 | 0 | 1 | 1 | 0 | 1 | 1 | 1 | 6 |
| ChiCTR1900026240 (Liu, 2022) | 1 | 0 | 1 | 1 | 0 | 1 | 1 | 1 | 6 |
| NCT03917966 (Wang, 2021) | 1 | 0 | 1 | 1 | 0 | 1 | 0 | 0 | 4 |
| NCT03985670 (Xing, 2021) | 1 | 1 | 1 | 1 | 2 | 1 | 1 | 1 | 9 |
| ESONICT-1 (Zhang, 2021) | 1 | 0 | 1 | 1 | 0 | 1 | 1 | 1 | 6 |
| Shen, 2021 | 1 | 0 | 1 | 1 | 0 | 1 | 1 | 1 | 6 |
| Yang, 2021 | 1 | 0 | 1 | 1 | 0 | 1 | 1 | 1 | 6 |
| SIN-ICE (Duan, 2021) | 1 | 0 | 1 | 1 | 0 | 1 | 1 | 1 | 6 |
| NICE (Liu, 2020) | 1 | 0 | 1 | 1 | 0 | 1 | 0 | 0 | 4 |
| KEEP-G 03 (Gu, 2020) | 1 | 0 | 1 | 1 | 0 | 1 | 1 | 1 | 6 |
| Zhang, 2020 | 1 | 0 | 1 | 1 | 0 | 1 | 1 | 1 | 6 |
| Li, 2020 | 1 | 0 | 1 | 1 | 0 | 1 | 1 | 1 | 6 |
| FRONTiER | 1 | 0 | 1 | 1 | 0 | 1 | 1 | 1 | 6 |


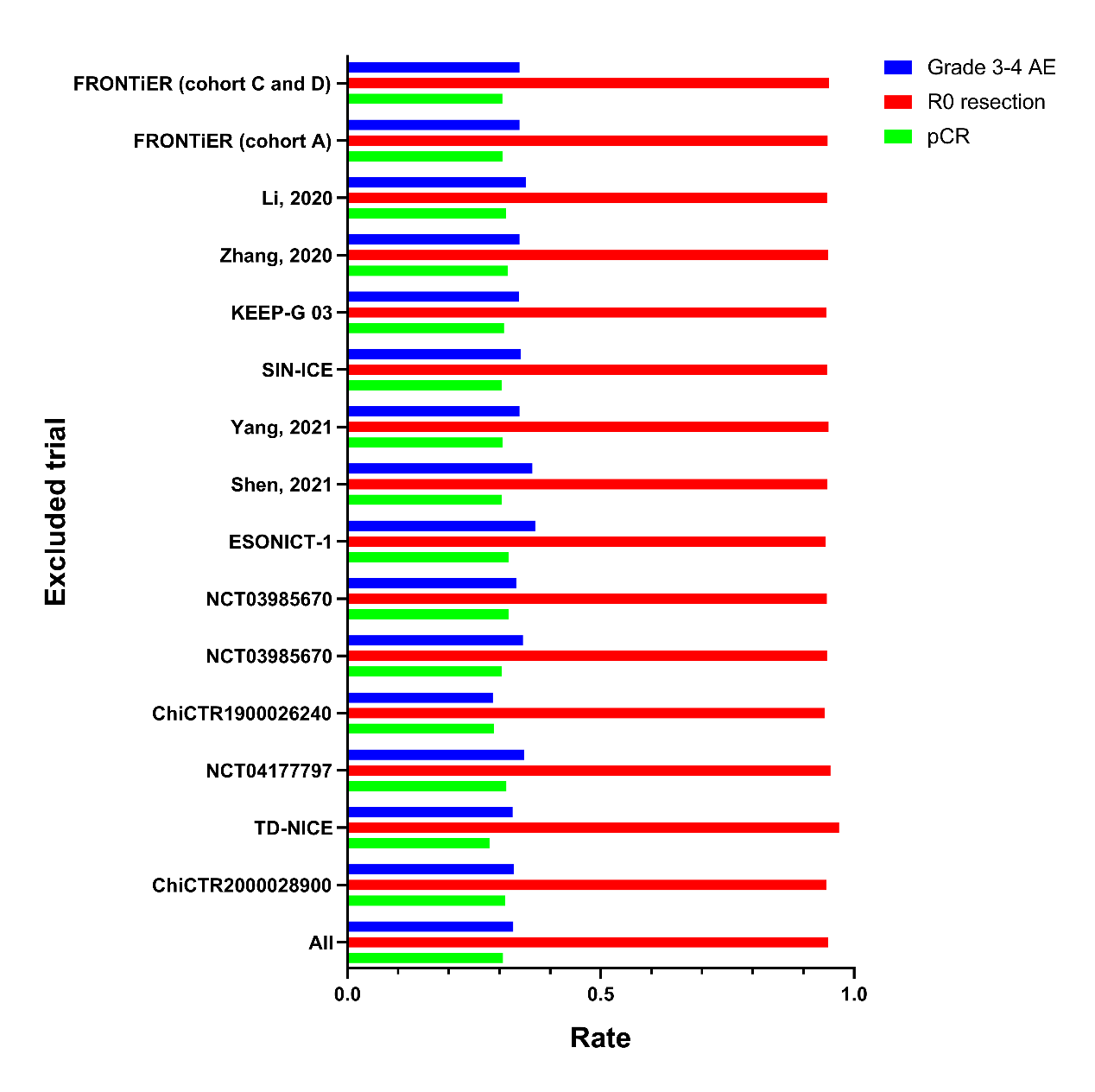


**Supplemental Figure 1.** The sensitivity analysis of 13 trials with neoadjuvant immunochemotherapy.
